# Cytotoxicity of rhein, the active metabolite of sennoside laxatives, is reduced by multidrug resistance-associated protein 1

**DOI:** 10.1038/sj.bjc.6600255

**Published:** 2002-05-06

**Authors:** B A P van Gorkom, H Timmer-Bosscha, S de Jong, D M van der Kolk, J H Kleibeuker, E G E de Vries

**Affiliations:** Department of Gastroenterology, University Hospital, PO Box 30.001, 9700 RB Groningen, The Netherlands; Department of Medical Oncology, University Hospital, PO Box 30.001, 9700 RB Groningen, The Netherlands

**Keywords:** anthranoids, multidrug resistance, apoptosis, colon, carcinogenesis

## Abstract

Anthranoid laxatives, belonging to the anthraquinones as do anthracyclines, possibly increase colorectal cancer risk. Anthracyclines interfere with topoisomerase II, intercalate DNA and are substrates for P-glycoprotein and multidrug resistance-associated protein 1. P-glycoprotein and multidrug resistance-associated protein 1 protect colonic epithelial cells against xenobiotics. The aim of this study was to analyse the interference of anthranoids with these natural defence mechanisms and the direct cytotoxicity of anthranoids in cancer cell lines expressing these mechanisms in varying combinations. A cytotoxicity profile of rhein, aloe emodin and danthron was established in related cell lines exhibiting different levels of topoisomerases, multidrug resistance-associated protein 1 and P-glycoprotein. Interaction of rhein with multidrug resistance-associated protein 1 was studied by carboxy fluorescein efflux and direct cytotoxicity by apoptosis induction. Rhein was less cytotoxic in the multidrug resistance-associated protein 1 overexpressing GLC4/ADR cell line compared to GLC4. Multidrug resistance-associated protein 1 inhibition with MK571 increased rhein cytotoxicity. Carboxy fluorescein efflux was blocked by rhein. No P-glycoprotein dependent rhein efflux was observed, nor was topoisomerase II responsible for reduced toxicity. Rhein induced apoptosis but did not intercalate DNA. Aloe emodin and danthron were no substrates for MDR mechanisms. Rhein is a substrate for multidrug resistance-associated protein 1 and induces apoptosis. It could therefore render the colonic epithelium sensitive to cytotoxic agents, apart from being toxic in itself.

*British Journal of Cancer* (2002) **86**, 1494–1500. DOI: 10.1038/sj/bjc/6600255 www.bjcancer.com

© 2002 Cancer Research UK

## 

Anthranoids are a group of substances with laxative action, including, amongst others, sennosides with their active metabolites rhein and rhein anthrone, aloe emodin and the synthetically produced danthron. Especially sennosides are commonly used as self-medication for constipation and chronic use of these laxatives has been associated with the development of *pseudomelanosis coli* (Steer and Colin-Jones, 1975). This condition is characterised by a brownish pigmentation of the colonic mucosa and is mostly regarded as a harmless phenomenon. *Pseudomelanosis coli* however, has been associated with an increased risk of colorectal cancer (Siegers *et al*, 1993). In addition, a single high-dose of sennosides was shown to induce an increase in proliferative activity of colonic epithelial cells (Kleibeuker *et al*, 1995), which is generally considered to be one of the first steps in colorectal carcinogenesis (Preston-Martin *et al*, 1990). Furthermore, several *in vitro* and animal studies have demonstrated mutagenic (Westendorf *et al*, 1990), genotoxic (Westendorf *et al*, 1990; Brown, 1980) and carcinogenic (Mori *et al*, 1985) effects of different anthranoid laxatives.

Anthranoid laxatives belong to the anthraquinones (Figure 1

**Figure 1:**
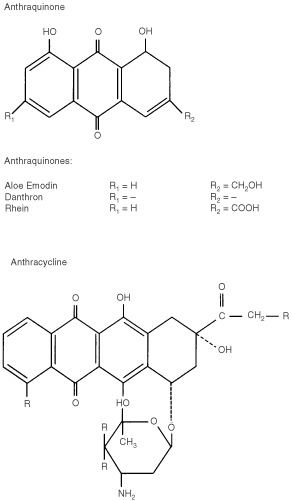
Structural formulas of anthranoid laxatives and anthracycline.

), a group of chemicals which also includes the cytotoxic anthracyclines used in cancer treatment. Anthracyclines exert their cytotoxicity by inhibition of topoisomerase II, intercalation of DNA and formation of free radicals (Stewart and Ratain, 2001). Thus leading to DNA damage and finally to induction of (apoptotic) cell death (Gamen *et al*, 1997). Repeated exposure of tumour cells to anthracyclines can lead to resistance for both anthracyclines and other natural product drugs with different chemical structure and mechanism of action (Goldstein, 1996), a phenomenon which is called multidrug resistance (MDR). Mechanisms contributing to MDR for anthracyclines include alterations in levels and/or activity of topoisomerase II (topo II) (Deffie *et al*, 1989) and increased expression of the drug efflux pumps P-glycoprotein (P-gp) (Hu *et al*, 1995) and of the glutathione dependent multidrug resistance-associated protein_1_ (MRP_1_) (Breuninger *et al*, 1995). P-gp and MRP_1_ drug efflux pumps are also expressed in normal colonic epithelium at low to intermediate levels (Chaman *et al*, 1996). They are probably involved in the protection of colonic epithelial cells against damage induced by xenobiotics (Shen *et al*, 1996). As anthranoid laxatives are also ‘natural products’ and chemically related to anthracyclines they are likely to be substrates for MDR mechanisms. The toxicity of anthranoid laxatives might, apart from their direct cytotoxic activity, be related to the effect of the laxatives on the defence mechanisms of the colonic epithelium making it more susceptible to other toxic agents.

In the present study the interference of anthranoids with the different mechanisms of MDR was studied in panels of related tumour cell lines expressing these mechanisms in different ways. In order to confirm anthranoid cytotoxicity apoptosis induction was studied in cell lines of different origin and DNA intercalation, as a possible mechanism of action, was determined in plasmid DNA.

## MATERIALS AND METHODS

### Chemicals

Rhein (9,10-dihydro-4,5-dihydroxy-9,10-dioxo-2-anthracenecarboxylic acid), aloe emodin (1,8-Dihydroxyl-3-(hydroxymethyl)-anthraquinone, danthron (1,8-Dihydroxyanthraquinone), MTT (3-(4,5-dimethylthiazol-2-yl)-2,5-diphenyltetrazoliumbromide), DL-buthionine-[S,R]-sulphoximine (BSO) and protease XXIV were purchased from Sigma (St Louis, MO, USA). Roswell Park Memorial Institute (RPMI) 1640 medium, foetal calf serum (FCS), Dulbecco's modified eagle (DME) and Ham's F12 (HAM) media were obtained from Life Technologies (Paisley, UK), dimethyl sulphoxide (DMSO) and acridine orange from Merck (Darmstadt, Germany). Rhein, aloe emodin and danthron were dissolved in DMSO. Doxorubicin was purchased from Pharmacia UpJohn (Woerden, The Netherlands) and vincristine sulphate-TEVA from Abic Ltd (Netanya, Israel). Carboxy fluorescein diacetate was obtained from Molecular Probes (Leiden, The Netherlands). Agarose I for electrophoresis was purchased from Amresco (Solom, Ohio) and ethidium bromide from Serva (Heidelberg, Germany). Dr Ford-Hutchinson, Merck Sharp, Canada kindly provided MK571.

### Cell lines

Table 1

**Table 1:**
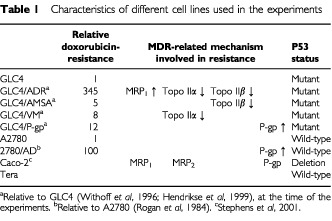
Characteristics of different cell lines used in the experiments

presents the cell lines used with their specific characteristics, according to which they were selected for the experiments. The first panel consists of GLC4, GLC4/ADR, GLC4/VM, GLC4/AMSA and GLC4/P-gp. GLC4 is a human small cell lung carcinoma cell line and, by ongoing incubation, GLC4/ADR is its at this moment 345-fold *in vitro* acquired doxorubicin resistant subline. Resistance in the GLC4/ADR is due to MRP_1_ overexpression and to a reduced Topo II activity (Zaman *et al*, 1993; De Jong *et al*, 1991). GLC4/ADR is cultured with 1.2 μM doxorubicin twice weekly. In the *in vitro* acquired 3-fold amsacrine-resistant subline GLC4/AMSA and the 20-fold teniposide-resistant subline GLC4/VM of GLC4, resistance is due to reduced Topo IIβ and Topo IIα expression respectively without overexpression of MRP_1_ or P-gp (Withoff *et al*, 1996). The GLC4/P-gp subline has a 325-fold resistance to vincristine compared to GLC4. GLC4/P-gp was obtained after infection of GLC4 cells with an MDR_1_-gene-carrying retrovirus (Hendrikse *et al*, 1999). GLC4/P-gp cells were cultured twice weekly with 50 nM vincristine sulphate to retain the transfected MDR_1_ gene.

The second panel of cell lines includes A2780, an ovarian carcinoma cell line, and 2780/AD its 100-fold *in vitro* acquired doxorubicin resistant P-gp overexpressing subline (Rogan *et al*, 1984). 2780/AD cells are cultured with 2.0 μM doxorubicin twice weekly. In addition two unrelated cell lines representing extreme inherent drug resistance and drug sensitivity, respectively, are used: the Caco-2 cell line, derived from a colon carcinoma (Fogh *et al*, 1977), and the N-Tera 2/D1 (Tera), an embryonal carcinoma cell line derived from a testicular tumour (Andrews *et al*, 1984).

All cell lines are routinely cultured in RPMI medium with 10% FCS. For the cell lines cultured in the presence of drugs, cells were cultured in drug-free medium for at least four passages before the start of the experiments.

### Screening of anthranoid laxatives by cytotoxicity profile

The microculture tetrazolium (MTT) assay was used for determination of drug cytotoxicity as described earlier (Timmer-Bosscha *et al*, 1989). In 96-well-plates (Nunc, Life Technologies, Paisley, UK), in 0.1 ml medium consisting of 40% HAM's F12, 40% DME and 20% FCS different numbers of cells were used for each cell line in order to assure linearity of the assay. For GLC4, GLC4/AMSA, GLC4/VM and for Caco-2 (in 0.2 ml) 3750 cells, for GLC4/ADR and GLC4/Pgp 10 000 cells, for A2780 1250 cells, and for 2780/AD 5000 cells per well were incubated with increasing concentrations of rhein, aloe emodin or danthron. Plates were kept at 37°C in a humidified atmosphere with 5% CO_2_. After 4 days incubation, 20 μl of MTT solution (5 mg MTT ml^−1^ phosphate buffered saline (PBS), (136 mM NaCl, 2.5 mM KCl, 6.5 mM Na_2_HPO_4_.2H_2_O, 1.5 mM KH_2_PO_4_, pH 7.2) was added. After 3.75 h incubation the plates were centrifuged (15 min, 180 g), supernatant was aspirated, 200 μl 100% DMSO added and extinction read at 520 nm (Timmer-Bosscha *et al*, 1989). The mean concentration that caused 50% cell kill (IC_50_) was determined in at least three independent experiments each performed in quadruplicate. The cytotoxicity assay with aloe emodin and danthron did not discriminate between GLC4 and GLC4/ADR or between A2780 and 2780/AD. Therefore these drugs were not studied further in the other cell lines.

### The role of drug efflux mechanisms, modulation of cytotoxicity

In the presence or absence of MRP_1_-inhibitor MK571 (50 μM) rhein cytotoxicity was determined in GLC4 and GLC4/ADR. To determine the glutathione-dependency of rhein cytotoxicity (Müller *et al*, 1994), GLC4 and GLC4/ADR cells were preincubated with the glutathione synthesis inhibitor BSO, at a concentration of 25 μM 24 h before testing, which was shown before to reduce cellular glutathione to levels below 0.1 μg glutathione mg^−1^ cellular protein (Meijer *et al*, 1987). The same experiment was performed with doxorubicin to determine efficiency of BSO preincubation. As glutathione synthesis is probably only inhibited as long as BSO is present, experiments were repeated with 4 h incubation of GLC4 and GLC4/ADR cells with rhein or doxorubicin, immediately following glutathione depletion. After 4 h, cells were washed three times with medium, then incubated for 4 days and analysed as described above.

### The role of drug efflux mechanisms, flow cytometric detection of carboxy fluorescein (CF) retention by rhein

Carboxy fluorescein diacetate (CFDA) is a nonfluorescent compound, which permeates the plasma membrane, and upon cleavage of the ester bonds by intracellular esterases, it is transformed into the fluorescent anion CF, which is a specific substrate for MRP_1_. The efflux of CF can be blocked specifically by the MRP_1_-inhibitor MK571 (Van der Kolk *et al*, 1998). The effect of rhein coincubation on cellular CF efflux was studied in GLC4 and GLC4/ADR. Of each cell line 5×10^5^ cells were incubated in RPMI medium for 20 min at 37°C with CF 0.5 μM or vehicle only, or combined with 15 μM or 60 μM rhein, or with 50 μM MK571 as a control. Cells were washed with ice-cold RPMI medium and then resuspended in RPMI medium 37°C without CF but with rhein 15 or 60 μM or MK571 and kept for 1 h at 37°C to allow for CF efflux or CF efflux-blocking. Efflux was stopped by pelleting the cells and adding ice-cold RPMI medium. Fluorescence of CF was analysed with a FAC-Star flow cytometer (Becton and Dickinson, Sunnyvale, CA, USA) equipped with an argon laser. The CF fluorescence of 10 000 events was logarithmically measured at a laser excitation wavelength of 488 nm through a 530 nm band-pass filter. The logarithmically acquired signals were converted into linear values and expressed as relative fluorescence units using the Winlist 2.0 program (Verity Software House, Inc., Topsham, ME, USA). Results are expressed as CF retention by the cells relative to the values found for CFDA plus MK571 coincubation. All experiments were performed in triplicate.

### Direct cytotoxicity of anthranoid laxatives, induction of apoptosis by rhein

To study the role of apoptosis susceptibility in anthranoid cytotoxicity, 10^6^ cells of GLC4, GLC4/ADR, Caco-2 and Tera cell lines were incubated with 100 μM rhein, and plated in PETRI dishes (6 cm diameter) in RPMI 1640 medium with 10% FCS. The GLC4 and GLC4/ADR were chosen because of different, probably efflux related, rhein induced cytotoxicity. As controls the Caco-2 a (colon) carcinoma cell line with a very low susceptibility to drug-induced apoptosis and Tera (embryonal carcinoma cell line) with a high propensity to go into apoptosis were used. In the Caco-2 cell line MRP_1_, P-gp and MRP_2_ (Buchler *et al*, 1996) are expressed (Stephens *et al*, 2001). In the Tera cell line no efflux pumps were detected (unpublished data). Besides, Caco-2 has a deleted and Tera a wild type p53 genotype, which may play a major role in their different drug sensitivities. After 6, 24, 48, 72 and 96 h acridine orange (1 mg ml^−1^) was added to distinguish apoptotic from vital cells by fluorescence microscopy (Olympus IM) (Evans and Dive, 1993). All experiments were performed in triplicate. Relative apoptosis induction was calculated: the per cent apoptotic cells after incubation with 100 μM rhein at a certain time point is divided by the per cent apoptotic cells in an untreated control sample at the same time point.

### Direct cytotoxicity of anthranoid laxatives, intercalation of DNA

Intercalation of anthranoids as a possible mechanism of action was studied by the unwinding of supercoiled plasmid DNA. Intercalation of a drug in supercoiled DNA leads to unwinding and this can be visualised by a reduced migration of plasmid DNA in an agarose gel (Hazlehurst *et al*, 1995). Supercoiled dimer of plasmid PBR322 was prepared from *Escherichia coli* strain HB101. Vials containing 3 μl PBR322, 9 μl tris-HCl-di-natrium-EDTA (TE) and 10 μl rhein, or aloe emodin, danthron or doxorubicin at different concentrations were incubated for 15 min at 37°C, 2 μl loading buffer was added to each vial and samples were put on a 1% agarose gel in tris-HCl-boric-acid-di-natrium-EDTA (TBE). Samples were run for 20 h at 20 V and stained afterwards with ethidium bromide (0.5 μg ml^−1^). DNA bands were visualised by transillumination with UV and photographed using the Imagemaster with Fuji Thermal film (Pharmacia Biotech, Roosendaal, The Netherlands). All experiments were performed at least in triplicate.

### Statistical analysis

The paired Student's *t*-test was used for statistical analysis. Only *P*-values <0.05 were considered significant.

## RESULTS

### Screening of anthranoid laxatives by cytotoxicity profile

The MTT assay was performed with three anthranoid laxatives in the GLC4, GLC4/ADR, A2780 and 2780/AD cells. For aloe emodin or danthron no difference in cytotoxicity was observed between GLC4 and GLC4/ADR or between A2780 and 2780/AD. Rhein however, was more toxic for the GLC4 than for GLC4/ADR and less toxic for the A2780 than for its doxorubicin-resistant subline 2780/AD (Table 2

**Table 2:**
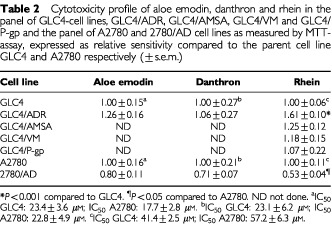
Cytotoxicity profile of aloe emodin, danthron and rhein in the panel of GLC4-cell lines, GLC4/ADR, GLC4/AMSA, GLC4/VM and GLC4/P-gp and the panel of A2780 and 2780/AD cell lines as measured by MTT-assay, expressed as relative sensitivity compared to the parent cell line GLC4 and A2780 respectively (±s.e.m.)

).

Resistance for rhein in GLC4/ADR can be related to either Topo IIα and/or β reduction or MRP_1_ overexpression. To investigate which of these mechanisms was most likely responsible for resistance to rhein, cytotoxicity of rhein was determined in the amsacrine resistant GLC4/AMSA and the teniposide resistant GLC4/VM, with resistance solely due to reduction of Topo IIβ and Topo IIα respectively. Cytotoxicity of rhein was the same in GLC4, GLC4/AMSA, and GLC4/VM (Table 2).

No difference in cytotoxicity of rhein could be demonstrated between GLC4 and the MDR_1_-transfected subline GLC4/P-gp (Table 2), whereas a distinct difference was seen in the cytotoxicity of vincristine (0.9±0.2 and 283.2^*^±45.1 respectively, ^*^P<0.002). To define Caco-2, one of the control cell lines in apoptosis induction, it was incubated with rhein in the MTT assay. The rhein IC50 was 64.3±11.6 μM (mean±s.e.m.) which is comparable to GLC4/ADR.

### The role of drug efflux mechanisms, modulation of cytotoxicity

Blockage of the MRP_1_-pump by coincubation with MK571 increased rhein toxicity in GLC4/ADR to the same level as that in GLC4 (Table 3

**Table 3:**
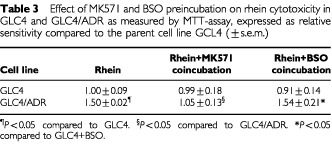
Effect of MK571 and BSO preincubation on rhein cytotoxicity in GLC4 and GLC4/ADR as measured by MTT-assay, expressed as relative sensitivity compared to the parent cell line GCL4 (±s.e.m.)

, Figure 2

**Figure 2:**
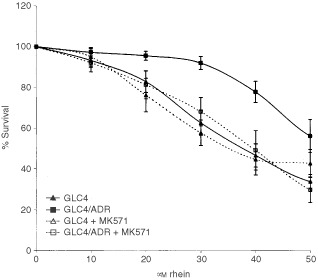
Survival curve of GLC4, GLC4/ADR, GLC4+MK571 and GLC4/ADR+MK571 after 4 days incubation with rhein in the MTT-assay. Increased toxicity (decreased survival) of rhein at IC_50_ in the GLC4/ADR cell line after inhibition of the MRP_1_-pump by MK571 (mean±s.e.m.). (*P*<0.05 compared to GLC4/ADR without MK571).

). After preincubation with BSO, followed by a 4 day, continuous, drug incubation there was no difference in rhein cytotoxicity in GLC4 or GLC4/ADR (Table 3), while doxorubicin cytotoxicity in GLC4/ADR increased (IC_50_ 6.8±1.9 and 5.0±5.3 μM (mean±s.e.m.) in GLC4/ADR and GLC4/ADR+BSO respectively, *P*<0.01). Experiments were repeated with 4 h drug incubation followed by a 4 day drug-free culture. Doxorubicin cytotoxicity increased during the 4 h incubation in GLC4/ADR after preincubation with BSO (IC_50_ 32.6±5.7 and 8.4±2.6 μM (mean±s.e.m.) in GLC4/ADR and GLC4/ADR+BSO respectively, *P*<0.01). However, due to poor solvability of rhein, no discriminating cytotoxic effect of this drug could be tested in this experiment.

### The role of drug efflux mechanisms, flow cytometric detection of CF retention by rhein

**Figure 3:**
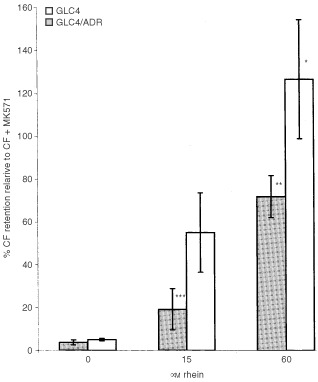
Cellular retention of CF in GLC4 and GLC4/ADR at different concentrations of rhein determined by flow cytometry. Retention of CF in GLC4 and GLC4/ADR in the presence of MK571 is defined as 100% retention and served as controls. CF retention in the presence of 15 μM or 60 μM rhein is expressed as relative to controls. In GLC4 complete retention of CF could be achieved at 60 μM rhein. In GLC4/ADR 72% CF retention is accomplished (mean±s.e.m.). *P<0.05 compared to CF in GLC4, **P<0.002 compared to CF in GLC4/ADR, ***P<0.05 compared to CF + Rhein 60 μM in GLC4/ADR.

To test whether rhein was also able to functionally affect the MRP_1_ pump, GLC4 and GLC4/ADR cells were loaded with the fluorescent MRP_1_ substrate CF with and without 15 or 60 μM rhein or 50 μM MK571 and efflux was allowed for 1 h. In GLC4 and GLC4/ADR CF efflux was blocked in the presence of MK571. To correct for inter-experimental fluctuations in the FACS analyses CF cell concentrations in the presence of MK571 were defined as 100% retention and served as controls. Figure 3

shows the relative retention of CF after incubation with rhein 15 μM or 60 μM compared to controls in both GLC4 and GLC4/ADR. CF efflux was blocked by coincubation with rhein at 60 μM in GLC4 (*P*<0.05) and in GLC4/ADR (*P*<0.002). There was a trend for higher CF efflux, and thus less retention, in the GLC4/ADR cell line compared to GLC4 at 15 (*P*=0.16) and 60 μM rhein (*P*=0.21).

### Direct cytotoxicity of anthranoid laxatives, induction of apoptosis by rhein

Figure 4

**Figure 4:**
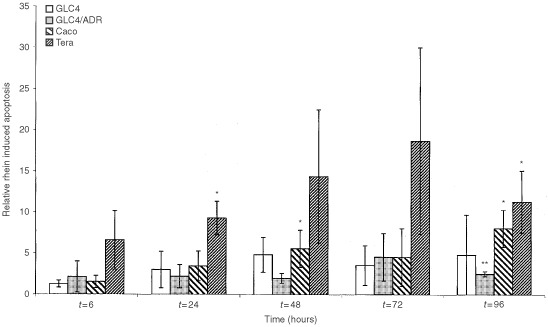
Relative rhein induced apoptosis determined by the per cent apoptotic cells after incubation with 100 μM rhein at a time point divided by the per cent apoptotic cells in an untreated control sample at the same time point. Cell lines used are GLC4, GLC4/ADR, Caco, and Tera. Time points are 6, 24, 48, 72 and 96 h after start of incubation. Values are expressed as mean±s.d.* significantly different from *t*=0, *P*<0.05, ** significantly different from *t*=0, *P*<0.001.

shows the relative rhein induced apoptosis. Apoptosis induction is found in the Tera cell line at 24 h and 96 h and a trend for apoptosis induction is also seen at 6 h (*P*=0.053). In the relative apoptosis resistant Caco-2 cell line at 48 h a significant induction of apoptosis is found. In GLC4/ADR after 96 h apoptosis was found while in GLC4 no significant induction was observed.

### Direct cytotoxicity anthranoid laxatives, intercalation of DNA

No intercalation of either rhein, aloe emodin or danthron could be demonstrated at concentrations of 1, 10, 100 and 10 000 μM, whereas with doxorubicin a shift of plasmid DNA was already seen at concentrations of 1 and 10 nM.

## DISCUSSION

Chronic use of anthranoid laxatives, such as senna, is considered to increase the risk of colorectal cancer (Siegers *et al*, 1993). In this study two routes which could be involved in this increase are studied, an effect of anthranoid laxatives on cellular efflux pumps reducing the physiological defence mechanisms of the colon epithelium and/or a direct cytotoxic effect of these agents on human cells.

With the use of related cell line panels this study demonstrates that the cytotoxicity profile of the active anthranoid laxative metabolite rhein is not homologue to that of doxorubicin in the panels of cell lines chosen. In the P-gp overexpressing cell line 2780/AD, the doxorubicin resistant subline of A2780, an increased sensitivity of 2780/AD for rhein was observed. This seems not to be related to increased expression of the P-gp drug efflux pump as in GLC4/P-gp no increase in rhein cytotoxicity was found compared to GLC4. Although the mechanism of this differential sensitivity of A2780 and 2780/AD for rhein is unclear, one could speculate that it could be due to the antimitochondrial activity of rhein (Verhaeren, 1980; Bironaite *et al*, 1992). Increased sensitivity to mitochondrial inhibitors has been shown in another doxorubicin resistant cell line (Bironaite *et al*, 1992) and the phenomenon of enhanced toxicity in 2780/AD has been observed before for a number of resistance modifying agents (Schuurhuis *et al*, 1990). As colorectal tumours are known to exhibit high levels of the P-gp protein (Fojo *et al*, 1987) and are intrinsically resistant to drugs involved in MDR it could be interesting to investigate whether a resistance modifying effect of rhein exists in human P-gp overexpressing colon carcinomas. Based on our study in cell lines the effect is not expected to be mediated through P-gp. Besides P-gp, MRP_1_ is found in normal colonic epithelium (Chaman *et al*, 1996) and colorectal tumours (Filipits *et al*, 1997). This drug efflux pump most likely protects colonic epithelial cells against xenobiotics induced damage (Shen *et al*, 1996) and is involved in the extrusion of several, structurally unrelated cytotoxic drugs including anthracyclines (Paul *et al*, 1996). In this study, we have demonstrated for the first time that rhein is a substrate for the MRP_1_ drug efflux pump. Cytotoxicity of rhein was reduced in the MRP_1_-overexpressing cell line GLC4/ADR compared to GLC4. The influence of alterations in topoisomerase II levels on this phenomenon was ruled out by experiments showing similar cytotoxicity of rhein in GLC4 compared to GLC4/AMSA and GLC4/VM, with reduced levels of topoisomerase IIβ and IIα respectively. The degree of rhein resistance does not seem very high. However, in the same cell line only a 4.4-fold resistance is found for vincristine (Cole *et al*, 1994), a well established MRP_1_-substrate of which the cytotoxicity is not affected by topoisomerase reduction.

Modulation experiments revealed a complete reversal of resistance of the GLC4/ADR cell line to rhein by coincubation of the cells with rhein and MK571, a leukotriene receptor antagonist (Paul *et al*, 1996) and MRP_1_ blocker.

Apart from cytotoxicity experiments efflux experiments with the fluorescent MRP_1_ substrate CF also showed that rhein is a substrate for MRP_1_. Rhein was, in a comparable concentration to MK571 (Van der Kolk *et al*, 1998), a blocker of CF efflux.

Transport function for several drugs of MRP_1_ is dependent on cellular gluthatione levels (Versantvoort *et al*, 1995). Cellular depletion of glutathione can be achieved by preincubation with BSO, a substance that specifically inhibits gluthathione synthesis in cells (Meister, 1991). It was previously shown that preincubation of GLC4 and GLC4/ADR cells with 25 μM BSO for 24 h reduced GSH levels effectively (Meijer *et al*, 1987). In this study we confirmed this by the increased toxicity of doxorubicin in GLC4/ADR cells. However, no effect of BSO preincubation on rhein toxicity was found. Although MRP_1_ has repeatedly been reported either to transport glutathione conjugates itself or to activate an endogenous glutathione-conjugate pump (Müller *et al*, 1994), it is not clear whether glutathione conjugation or co-transport are always needed for MRP_1_ activity (Paul *et al*, 1996; Broxterman *et al*, 1995). Recently, it was shown that cellular glutathione depletion did not affect MRP_1_-dependent extrusion of the acetoxymethylester of calcein or free calcein either (Holló *et al*, 1996). In contrast to our study, in an *in vitro* study with rat hepatocytes increased rhein cytotoxicity was found after 5 h preincubation with 0.5 mM BSO (Bironaite and Öllinger, 1997). Although it can not be excluded that the 20-fold higher BSO concentrations used in that study compared to our study is toxic in itself for rat hepatocyte cells, evaluation of any short-lasting (4 h) effect of 24 h BSO preincubation could not be determined in our experimental design, as the solvability of rhein did not allow high enough concentrations to induce cytotoxicity.

Induction of carcinomas by anthranoid laxatives have been demonstrated in animal studies (Mori *et al*, 1985) and mutagenic and genotoxic effects were described in bacterial strains (Westendorf *et al*, 1990; Brown and Brown, 1976). Mutations occurred predominantly in those strains sensitive to frame-shifting mutagens (Brown and Dietrich, 1979), which suggest that anthranoids might achieve DNA-damage probably by intercalation. In the present study no DNA-intercalating capacity of rhein, aloe emodin or danthron could be demonstrated in the bacterial plasmid DNA PBR322.

Anthranoid laxatives have been shown to induce apoptosis of colonic epithelial cells in man within 6 h after ingestion (Van Gorkom *et al*, 2001). Animal studies also demonstrated increased apoptosis 6 h after danthron administration (Walker *et al*, 1988). In our *in vitro* study, although little apoptosis was found in the GLC4 and GLC4/ADR cell lines rhein induced apoptosis in the control cell lines. These different effects are more likely to be cell type than resistance related. In the apoptosis sensitive cell line Tera the highest apoptosis induction was found. Caco-2, a colon carcinoma cell line which is relatively insensitive to apoptosis-induction by doxorubicin, readily underwent apoptosis induction after 48 h incubation with 100 μM rhein. This concentration is below the concentration reached in the colonic lumen after therapeutic doses of anthranoid laxatives used as a bowel preparation for diagnostic procedures (Schörkhuber *et al*, 1998). Single doses of 0.15 g sennosides corresponding to 0.5 mmol rhein anthrone in approximately 2 litre colonic contents yield concentrations of about 250 μM.

In conclusion, this study demonstrates that rhein, the active metabolite of sennoside laxatives, is a substrate for the MRP_1_ drug efflux pump and is a cytotoxic agent capable of inducing apoptosis. This cytotoxicity is neither topoisomerase II related nor a result of DNA intercalation. The capacity to extrude the anthranoid laxative may be important to protect the epithelium. Evaluation of colonic MRP_1_ levels in chronic users of anthranoid laxatives can be of interest. It may well be that the risk to develop colon cancer in anthranoid laxative users is negatively related to MRP_1_ expression. In addition also mutations or polymorphisms in MRP_1_ may play a role.
